# Research on the design of somatosensory interactive games for autistic children based on art therapy

**DOI:** 10.3389/fpsyt.2023.1207023

**Published:** 2023-10-06

**Authors:** She Huili, Cao Xiaolin, Guo Guangsen, Jiang Yu, Liu Yu, Zhang Wenpei

**Affiliations:** Department of Art and Design, Anhui University of Technology, Maanshan, Anhui, China

**Keywords:** autism, somatosensory game, intervention, art therapy, game therapy

## Abstract

There is no targeted drug treatment for autistic children. Educational intervention and rehabilitation are the main ways to improve the ability development of autistic children. However, there are great differences in the individual symptoms and abilities. It is an urgent need for educators, practitioners and parents of autistic children to find effective ways to improve their cognitive, social and motor abilities. The author cooperates with the therapist to study and design the somatosensory interaction game for autistic children, which is a formal attempt of art therapy on the treatment tool, with the purpose of studying an effective and safe art intervention method. In order to verify the effect, the author recruited 26 children with mild or moderate autism for a six-week empirical evaluation, and the participants were randomly assigned. Among them, 13 participants participated in the art therapy of the proposed somatosensory game group (the experimental group), and 13 participants participated the traditional picture book group (the control group) that improved the emotion, cognition and skills of children, and the design style was more popular with children. The aesthetic design in the picture book could have an intervention effect on the participants, and its artistic effect could serve as an effective reference for the interface design of the game group. The results showed that the two groups had a significant impact on the participants in different aspects. The game group improved not only in terms of concentration and special ability, but also in terms of physical coordination and activity enhancement, it is inferred that the core factors of game therapy are sub-intervention, interaction and feedback, icon design and color matching system; the picture book group has significant improvement in interpersonal relationship guidance and language learning and communication. The results show that it is necessary to take more comprehensive and richer preliminary research on the development of treatment products for autistic children. Because picture book education requires children’s initial concentration, it is found that picture book therapy has high requirements for teachers’ classroom control and relatively strict requirements for picture book content, and play therapy can be a good complement to these problems.

## 1. Introduction

Autism spectrum disorder (ASD) is a syndrome with severe psychological development disorders in social communication, language communication, emotional regulation, behavior and action (1). According to the statistics of the Health Commission of China in 2019, autistic children accounted for 36.9% of children with mental disabilities, which is the primary cause of mental disability in children (Report on the development of autism education and rehabilitation industry in China). However, despite the high incidence rate of autism, the rehabilitation of autism is not ideal. General, high-quality treatment for autism requires professional training courses, which require not only high costs, but also experienced trainers. More importantly, the treatment of autism is not only in the course, but also requires adequate training after course. But for developing countries or ordinary families, it is difficult to meet this. Therefore, it has become an important trend to explore interventions that can be operated at home after course, with a higher degree of standardization and lower operating costs. Art therapy for autistic children came into being under this trend.

Art therapy is a healing process when art forms act as a medium in the field of psychotherapy. Research shows that some art forms such as somatosensory interactive games and picture book reading are deeply loved by autistic children, which can not only improve their symptoms, but also promote psychological development and improve social functions (2), these may become a new research direction of art therapy. As a medium of expressive art therapy, picture books can be called “relationship carriers” to connect, deepen and extend relationships. They can play an active role in children’s psychotherapy, psychological counseling and psychotherapy. However, picture book therapy has some requirements for children’s initial concentration. In the experiment, it was found that there are high requirements on teachers’ classroom control, and on content of picture book, but game therapy can well complement these problems.

Game therapy refers to a psychological healing method that uses games as a healing medium and carrier, allowing children to express and reveal their emotions, experiences, and behaviors in games, in order to achieve growth and development. The purpose is to help children vent negative emotions, develop intelligence and social skills, and form a perfect personality through play activities. It has been proved by practice that game therapy has obvious effect on the treatment of children’s psychological disorders, and has a positive impact on anxiety, autism, depression, aggressive behavior, mental trauma, emotional adjustment disorders, cognitive Learning disability, etc. Researchers have also carried out game therapy practices from different disciplines: constain have developed the “FRIDA” computer framework, a guide for agile development of accessible software for users of Autism spectrum Disorders (ASD) as a tool for strengthening emotional and social skills in the treatment of autism. Using this achievement can reduce development time by 94% and improve availability levels by over 90%, thereby promoting agile design of accessible applications. Chen attempted to apply somatosensory interaction technology to facial expression recognition in children with ASD in 2005. Later, researchers at the Hopkins Institution developed a children’s social skills intervention game system called Face Say, which consists of somatosensory interaction technology. Patel et al. designed the avatar virtual character Me Emo to improve children with social problems. By observing the facial expression of Me Emo, they judged the needs of Me Emo and gave corresponding help, so that children with mental disorders can understand the emotions expressed by others (3). This study found that ASD children communicate more flexibly and naturally with virtual characters than with real characters, and have better language expression ability, children are more willing to engage in social activities in virtual environments. Iris showed the results of psychological problems between parents and children caused by the use of game therapy intervention, and the results proved that the emotional problems and contradictions between parents and children in families were significantly alleviated under the intervention of game therapy (4). Compared with other therapies, gametherapy can bridge the emotional distance between parents and children, enhance mutual understanding, and provide positive guidance for improving children’s emotional regulation disorders. The fun inherent in games is more likely to attract children’s attention. By intervening in children with cognitive impairment through games, it can achieve good improvements in children’s interest in learning and cognition, concentration, self-confidence, and expression ability. Research has found that the movement segments in games have a natural attraction for children with ASD and are not easily rejected by children with autism. The somatosensory interactive games used in this study can also create virtual environments, which not only have better security and targeting, but also have higher immersive potential (5).

## 2. Implementation of intervention mode

Due to the lack of quantitative research, art therapy has not been fully considered as an important intervention for autism (6), but in recent years, computer-based therapy has been proved to be effective in learning, cognitive and social aspects of children with ASD, and the corresponding computer games have become a relatively novel learning method (7, 8). This study intends to take the picture book reading activity with specific functions as the picture book group and the somatosensory game activity as the game group. Through the recruitment of autistic children for a randomized intervention experiment, this way of comparing the game group with the picture book group may also obtain more useful information (9), and ultimately provide help for designing a safe and targeted somatosensory interactive game, then explore a widely used somatosensory game intervention method. The game itself is just a medium of treatment, providing a warm and safe healing environment for children with ASD. Relying on the child’s inner self ability and effective intervention methods, it promotes problem-solving and recovery.

The intervention behavior of therapists in experiments generally takes the following forms: non directive, directive, and compromise forms, as well as selecting methods based on personal preferences (10) or other artistic methods applied to the psychological treatment of new adolescents (10–13). The research results indicate that using appropriate intervention techniques is effective for children with psychological problems (14). In this experiment, in order to obtain the net effect of the experimental operation, a non guided intervention approach was adopted, where the therapist only kept track and promoted the subjects (15), in order to objectively analyze various ability indicators of ASD children before and after the test, this process is entirely completed independently by ASD children. Although most of the somatosensory games used in the experimental process were case studies, in practical applications, the author encourages parents to intervene in the growth of children with autism, which has a strong impact on the healthy development of children. Parents use games to leverage the unique emotional advantages between parents and children, bringing them closer to the emotional expression styles of children with autism, invisibly increasing the learning opportunities of children with autism (16, 17). In addition, the virtual context built in the somatosensory interaction game also provides a more private communication environment for parents and autistic children to vent and heal their emotions, which can improve the construction of the somatosensory interaction game therapy system.

## 3. Method

The research procedures of this study are: recruitment of subjects, design of game therapy, implementation and analysis of intervention experiment.

### 3.1. Recruitment of subjects

This study was approved by the local special education school and there is no conflict of interest. Participants were recruited through an open on-campus recruitment process by the school. After receiving a complete explanation of the study, the guardian and participants agreed to participate and provide a written informed consent to release any potentially identifiable graphics and data contained in the text. The inclusion criteria of participants are: combination with medical identification, according to the degree of students’ teaching support, mild or moderate autism students, aged 7–12 years (see Table 1). In this study, the Children Autism Rating Scale (CARS) and the Design Assessment Sheet for somatosensory Interactive game therapy were required to be completed before the experiment, and the Behavior Checklist for Children with Autism (ABC) was completed in the teacher’s guidance during the test. Because there are great differences in individual symptoms and abilities of ASD children, it is a wise method to use the Assessment scale. In the experiment, 26 children were randomly divided into picture book group and game group for more detailed analysis, including focusing on eye contact, attention, body coordination and other aspects (18–20). The overall implementation framework is game therapy architecture – evaluation – game design – intervention design – empirical test. All the families were willing to participate in the experiment and reserved the right to withdraw at any time.

**Table 1:** Participant basic characteristics status.

A. Picture book group											
No	Gender	Age	Years of education	Interactive responses	Verbal communication	Emotional response	Somatic gaming experience	Other issues	Baseline test score		
									① 60	② 60	③ 62
1	F	10	3	Often	Nothing		NO	Stereotyped behavior	32	35	40
2	M	9	2.5	Often	Often	Unstable	YES		34	28	45
3	M	7	1	Often	Nothing	Stable	NO		30	31	38
4	M	8	3	Seldom	Often	Stable	YES	Finger-wrestling Hand-squeezing Basic play experience	31	33	42
5	M	11	3	Often	Often	Stable	NO	Switching light	34	30	41
6	M	7	1.5	Often	Often	Stable	NO		36	32	40
7	M	8	2.5	Seldom	Often	Stable	YES	Spinning	31	28	36
8	M	6	1.5	Often	Seldom	Stable	NO		35	32	38
9	M	9	3	Seldom	Often	Unstable	NO	Play games	32	30	40
10	M	8	2	Often	Seldom	Stable	NO		30	31	37
11	M	9	2.5	Often	Often	Stable	NO	Repetitive language	28	34	39
12	M	7	2	Often	Nothing	Stable	NO	Play games	31	35	40
13	M	8	2.5	Seldom	Often	Stable	YES	Repetitive language	33	31	42

B. Game group											
No	Gender	Age	Years of education	Interactive responses	Verbal communication	Emotional response	Somatic gaming experience	Other issues	Baseline test score		
									① 60	② 60	③ 62
1	F	11	3.5	Seldom	Often	Stable	YES	Open and close doors	36	30	41
2	M		2	Often	Often	Stable	NO		31	32	40
3	M	9	2.5	Nothing	Seldom	Stable	YES	Refuse contact with others	32	29	36
4	M	7	1.5	Often	Often	Stable	NO		34	32	37
5	M	10	2.5	Often	Often	Stable	NO	Repetitive language	30	31	39
6	M	9	2.5	Often	Often	Stable	NO		35	34	40
7	M	8	2	Often	Nothing	Unstable	YES	Stereotyped behavior	32	36	42
8	M	7	1.5	Nothing	Often	Unstable	YES	Run in circle	30	31	40
9	M	8	2	Often	Often	Stable	NO	Play games	28	35	45
10	M	9	2.5	Nothing	Seldom	Unstable	YES		35	35	38
11	M	8	2	Often	Often	Stable	NO	Repetitive language	32	33	42
12	M	8	2	Nothing	Often	Unstable	NO		30	31	45
13	M	7	1.5	Nothing	Nothing	Stable	NO	Wink, nod	35	32	47

### 3.2. Appeal and strategy of somatosensory games

Autism spectrum disorder has atypical social interaction and behavior, so autistic children need intensive intervention in children’s cognitive, emotional management, behavioral ability, social ability and motor ability (21), and exercise of these skills is considered essential for long-term independent life, over the years, various technologies have been developed (22–27). Due to the particularity of the symptomatic popular and the different characteristics of the basic abilities of the popular, this paper adopts the diversified game theory in the design of the somatosensory interactive game. For example, Freud’s psychoanalytic theory is used to make necessary mental catharsis for the emotional regulation of autistic children; In view of the weak social communication skills of autistic children, try to use Engstrom’s collective cultural game theory to design the game interaction part to create a good collective environment built by peers, parents, teachers and other caregivers (28). The flexibility and variability of somatosensory interaction games can be used as both peer interaction games in teaching and parent–child emotional games in families. It is an important therapeutic tool to strengthen the social behavior of autistic children and improve their social ability by establishing appropriate social game models.

In order to enhance the therapeutic effect of somatosensory game design products on children with autism, the team searched for corresponding theoretical basis for psychological therapy for the possible causes of ASD children, formulated specific and actionable elements for constructing somatosensory game therapy, and proposed corresponding game demands or strategies to improve the therapeutic framework (see Table 2).

**Table 2:** Theoretical basis and strategy of somatosensory game therapy.

Construction reasons	Theoretical basis	Construction elements	Appeal or strategy
Difficult to accept unfamiliar environments and groups	Theory of collective cultural games	Ecologically formed game groups	(1) Active gaming (2) Get along well with peers (3) Willing to play the role of ‘playmate’ (4) Accepting diverse social contexts
Emotions are difficult to control, and the demand for children’s safety in games increases	The relationship between game space design and the possibility of children participating in games	Virtual game scenarios and personalized game spaces	Simulate real-life scenarios The limited area of the venue ensures the safety of children with autism (3) Can create personalized spaces
The research subjects often lack abilities in one aspect, but have unique talents in another aspect	Game elements are important factors for children with autism to improve their cognitive ability, interpersonal communication, and emotional well-being	Controllable and imaginative game elements	Must meet the visual preferences of ASD children Suitable for the development of autistic children’s abilities, controllable situations, and highly imaginative game elements. Design types that align with the game itself (such as sports and cognitive).
The emotional intervention of the research object is a long-term and uninterrupted healing process, and flexibility, mobility, and gradual progress are the requirements of healing	Non guided play therapy	Staged process implementation arrangement	Clarify the rules for game implementation (2) Visual game elements can be used to determine game rules (3) Visual game elements can be used to determine the game rules and refer to the child’s healing status based on the game clearance process, as a basis for emotional therapy in the next stage of children.

### 3.3. Evaluation of the applicability of somatosensory games

To ensure that somatosensory interactive game therapy is effective in treating emotional disorders in children with autism, the research team conducted multiple assessments of the subjects before implementing the game intervention. Firstly, the emotional characteristics of the participants were identification. Through interviews and observation, the ways of children’s emotional expression were identified. The emotional expression of children was classified into solitary type and cathartic type. Secondly, the participants’ understanding of the general meaning of games was tested, and the evaluation was divided into three levels: no feeling, watching and trying. The social mode of games was set into three levels: personal, peer and family, etc., in order to fully grasp the participants’ support degree for game healing before the experiment. Thirdly, the preliminary game style, theme elements and game mode are determined by targeted design according to the participants’ preference for artistic style and social mode.

### 3.4. Content design of somatosensory games

**Figure 1:**
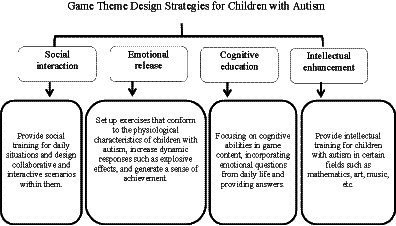
Game theme design strategies for children with ASD autism.

The content design of somatosensory interactive games for ASD children includes the selection of game themes, writing of game backgrounds, innovation of game gameplay, and planning of game levels. Prior to this, it is necessary to define user needs and the ultimate goal of the game. Due to the difficulty in establishing peer relationships among children with autism and the possibility of being excluded from the social environment (29), developing game skills and setting common game hobbies may be important channels for children with autism to establish social capital with their peers. The game design for children with ASD selects electronic game based somatosensory devices as the presentation medium, with the aim of improving the emotional disorders and social communication problems exhibited by children with psychological disorders through an immersive gaming environment. Through the analysis of game therapy theory and related cases in this article, it can be concluded that the problems exhibited by children’s psychological disorders may be one or multiple, therefore, the problems that ASD children need to solve are not just single problems. In this game classification setting, we mainly set the game into four categories: social interaction, emotional venting, cognitive education, and intellectual enhancement. For example, social interaction involves conducting social training on certain situations in daily life and designing collaborative interaction scenarios within them. Cognitive education focuses on improving cognitive abilities in game content, incorporating emotional questions from daily life and providing answers; The theme of emotional release involves setting up a certain amount of dynamic responses such as physical movements or explosive effects, generating a certain amount of visual stimulation; Intelligent theme development records and evaluates the talents of children with autism in a certain field (such as mathematics, art, music, etc.); Each type of game is designed with five aspects: entry into the game, game theme, game guidance, design methods, and game settlement. Enter the game, establish personal body data and game profile; Determine the game theme. Due to the limited abilities of children with autism, game design often tends to focus on thematic and specific skill training for educators, professionals, and families to adopt (30, 31) (see Figure 1).

### 3.5. The information architecture of somatosensory games

**Figure 2:**
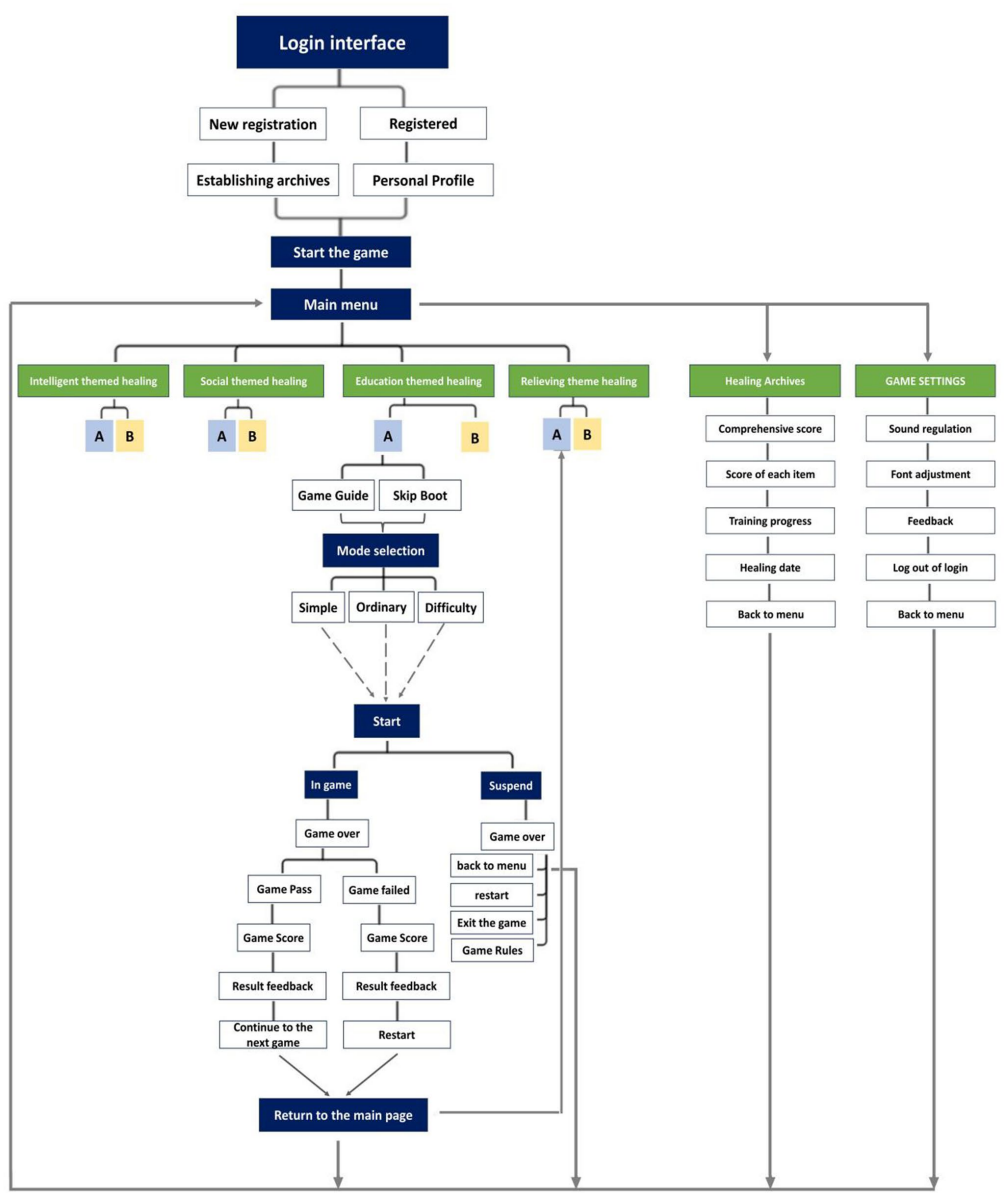
Information architecture of sensory interactive games.

The game design for ASD children incorporates the basic architecture of pre and post testing for the experimenter. The main menu of the game belongs to the first layer of the game information architecture, which is the entrance for children with autism to enter all operations in the game. The experimenter can create a personal profile here. In the main menu of the game, there are three main entrances: the healing theme entrance, the healing status file entrance, and the healing game settings entrance, the three major entrances are all the second level of the sensory game information architecture. This game features various themed games, and ASD children can choose according to their interests and hobbies. In order to make the game experience more comfortable and easy for users to understand, the game adopts a non blocking guidance design and a simple interface design. After the game is over, accompanying personnel can enter their personal profile and record the scores of the experimenter (see Figure 2).

### 3.6. ASD children’s game interface design

In order to provide ASD children with more intuitive experimental products, the art team designed two somatosensory interactive themed games, one for puzzle themed “Digital Maze” and the other for sports rehabilitation themed “Happy Kitchen,” to test the reactions of ASD children in these two abilities. Both games have designed theme interface styles, interface layout, game guidance signs, game direction, and reward mechanisms. These contents are designed with reference to the research findings of Whyte et al. (32) and Banire et al. (33) (see Table 3).

**Table 3:** Physical interaction game content.

Topic name	Game theme	Game dimension	Guidance method	Feedback mechanism	Style setting
Digital maze	Puzzle category Cognitive	Difficulty progress reward mechanism	Voice guidance	Success feedback Failure feedback Game End Feedback	Simple, cartoon, flat design, high-frequency element animal image
Happy kitchen	Sports Cognitive	Difficulty progress reward mechanism	Finger guidance	Success feedback Failure feedback Game End Feedback	Simple, cartoon, flat design, kitchen utensils, vegetable image

**Figure 3:**
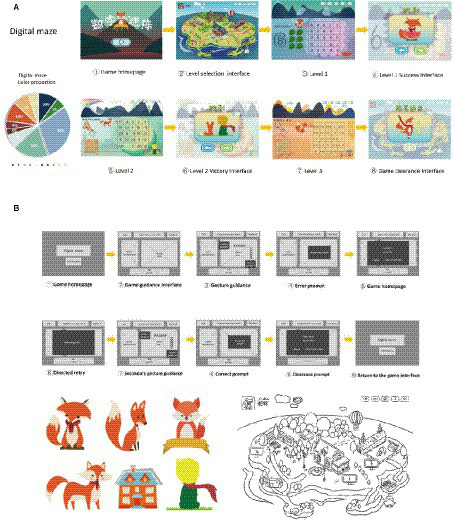
“Digital maze” interface, color proportion, guidance, element design. **(A)** “Digital maze” the flat home screen design blue green and cold colors. The ratio of cold and warm colors in the overall game is 7:3. The difficulty of each level is increasing. Different levels are configured with different building groups, and the pop-up frame flashes. **(B)** Non blocking guidance design: there are a total of 10 interface jump interactions in the interaction flowchart. Except for the gesture interface that needs to be guided by gestures and then automatically jump, other interfaces will automatically jump every 3 s. A menu bar will be designed above the interface, and text prompts will be displayed below. The left side of the interface is the game requirements, and the right side is designed with gesture guidance. The game area will be placed in the visual center.

**Figure 4:**
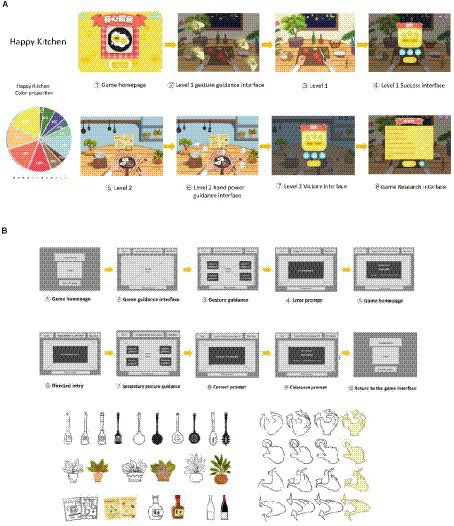
“Happy kitchen” interface, color proportion, guidance, element design. **(A)** “Happy kitchen”: the flat home screen is designed with high brightness value and increasing difficulty. The gesture guidance design is set for users to imitate. You can control the use of tableware through the handle, and attract attention by flashing the button. **(B)** Gesture guided animation is set at the visual center of the interface, there are many types of kitchen items, and they are guided to be used through gesture guidance, the rest are the same as Figure 3B.

Before the formal implementation of the design, the therapist and designer communicated fully, and the team tracked and observed the ASD children in the collaborating unit to ensure a thorough understanding of their artistic preferences for visuals. The team ultimately designed two somatosensory interactive games, with features such as simple and easy to understand visuals, cartoon graphics, flat design, and easy to operate signage. This is basically the same as the usability standards of Constain et al.’s research (30). Various images related to the theme game were identified in the screen, such as high-frequency animal images, maze fields, kitchen backgrounds, kitchen utensils, etc. (see Figures 3, 4).

Game guidance design is an important part of game Interaction design, which has an important impact on the game experience of autistic children and the effect of game therapy. In this design, the guided design process is divided based on the game information architecture and guided by the game scene. Both games adopt a non blocking guidance design, with a total of 10 interface jump interactions in the interaction flowchart. Except for the gesture interface that requires children with autism to complete gesture guidance and jump on their own, the rest of the interfaces automatically jump every 3 s (see Figures 3B, 4B).

In order to improve the acceptance of colors by ASD children when using games, a color test was conducted before the experimental design. The team randomly selected 12 ASD children from the list of participants for color preference testing, and displayed three sets of images with different colors, saturation, and brightness in sequence. Participants were asked to choose a color card of interest from each set of images, and the final results showed that among the 12 children, eight children chose cold tone color cards, seven children chose high brightness color cards, and nine children chose high saturation color cards. Therefore, it was preliminarily concluded that cold tone, high brightness, and high saturation colors are more likely to attract the attention of children with autism (see Table 4). In the upcoming game interface color matching, the team selected the colors preferred by children with autism, and in order to highlight the main visual image, added a color interface with a contrast between cold and warm. In terms of color details configuration, the two games combine some scholars’ research on the color system preferred by ASD children (34). “Digital Maze” mainly features cold tones, with the protagonist Fox using warm tones, and “Happy Kitchen” mainly features bright gray contrast, with the main objects being pure tones. In the screen, there are also some scenes that assist ASD children in understanding, such as the elements of the forest in “Digital Maze,” the kitchen elements in “Happy Kitchen” have undergone significant degradation adjustments in color brightness and purity, and have been placed in non visual center positions in the panel design to avoid elements being too prominent and interfering with children’s attention (see Figures 3A, 4A).

**Table 4:** The results of the ASD children’s color preference test, the overall preference is for cool, high brightness, and high saturation colors, which are attracted by shiny colors.

Color research for testers testers												
Testers	1	2	3	4	5	6	7	8	9	10	11	12
Color style												
Cool tone	√		√			√	√	√		√	√	√
warm-toned		√		√	√				√			
High brightness	√		√	√		√			√		√	√
Low brightness		√			√		√	√		√		
High saturation		√		√	√	√	√	√	√	√		√
Low saturation	√		√								√	

**Figure 5:**
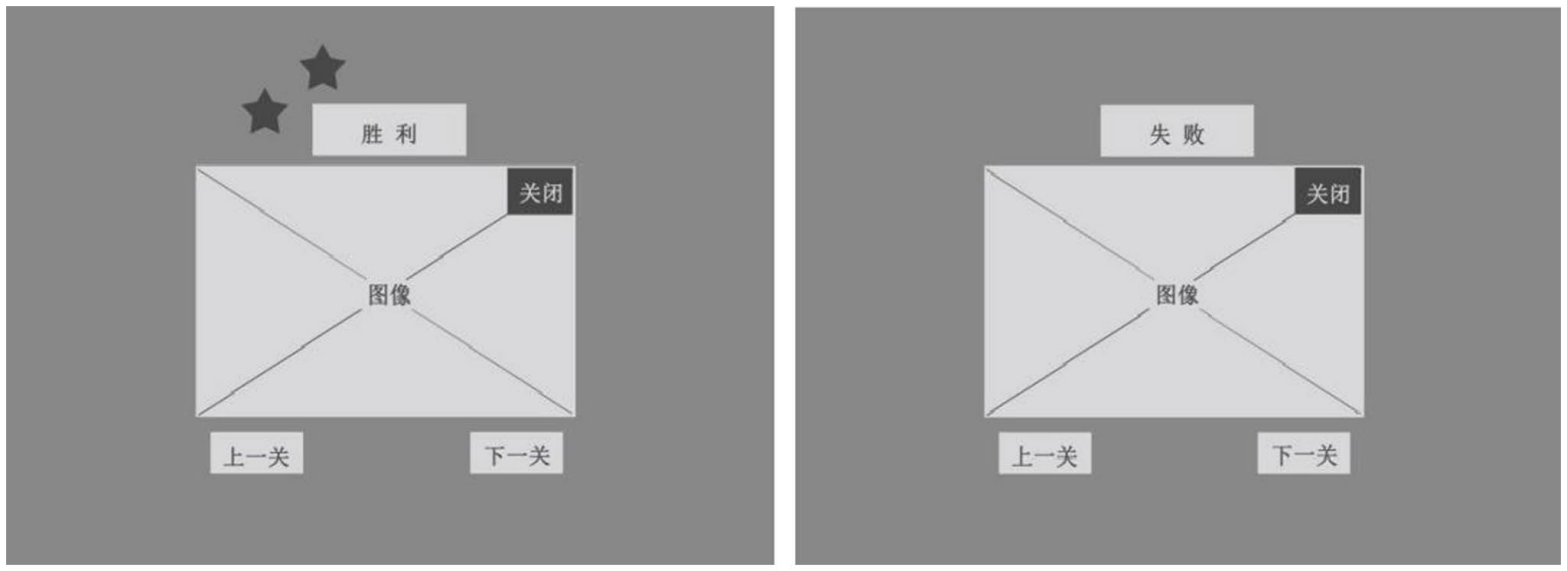
Design of “Reward mechanism” and “Comfort mechanism.”

Some studies have shown that built-in reward systems in games may have a more motivating effect than other types of educational interventions (35). In the two game results designed by the author, a reward mechanism for “victory” and a consolation mechanism for “failure” are generated based on the player’s experience in game healing. The reward mechanism will present a visual dynamic effect of “star twinkling” to children with autism, and additional token rewards will be given to the player (see Figure 5).

Considering that customized rewards may affect the participants’ subsequent reactions and make the results more complex, we did not consider customized rewards in this experiment. However, in the future, we may provide a comprehensive customized reward based on the participants’ final scores.

### 3.7. Feedback design for somatosensory game

**Figure 6:**
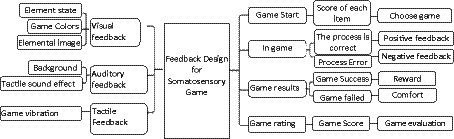
Feedback design for somatosensory game.

The feedback design of somatosensory games in the treatment of ASD children is a diverse feedback design tailored to their psychological and physiological characteristics. The interaction design of somatosensory games for children with autism pays great attention to the structure of feedback, evaluation, and reward mechanisms, providing positive affirmation to their gaming behavior, stimulating positive emotions, and helping them improve their emotional regulation and social skills. The feedback design of ASD children’s somatosensory games is carried out through three channels: visual feedback, auditory feedback, and tactile feedback. Feedback design includes four aspects: game start, game start, game results, and game evaluation during the game process (see Figure 6).

### 3.8. Reading therapy of picture books

**Figure 7:**
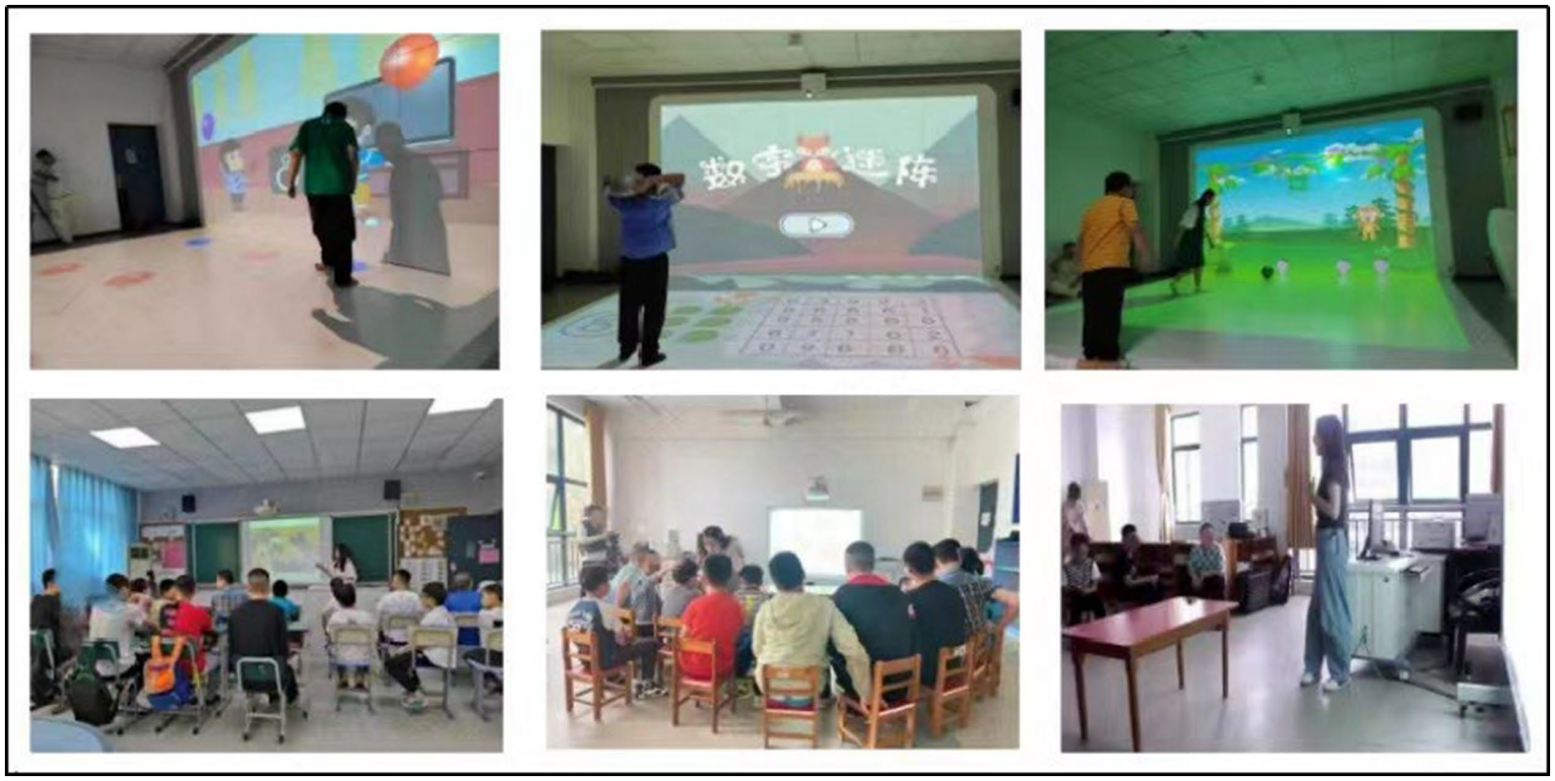
At the intervention site of body feeling interactive games and picture book activities, the top row of pictures shows children with ASD playing physical games, and the next row of pictures shows children with ASD learning from picture books. Non guiding interventions are adopted in body feeling interactive games, advocating for independent completion by participants. Picture books mainly focus on collective activities with multiple people and emphasize communication with participants.

Children with ASD usually exhibit hyperactivity disorder, inattention, resistance and dependence behavior (36), and some oral or written information is difficult for them to understand during intervention activities (37). Reading therapy provides a way to use children’s picture books to achieve social, emotional and behavioral goals (38). The picture of the designed picture book is relatively specific and clear, which is very different from the ordinary picture book. The picture book language is concise and the content is continuous, which enables autistic children to maintain better visual attention to the picture book content (39), and helps improve their concentration, communication ability and imagination (40). In this experiment, three types of picture books were selected for picture book teaching: emotion control, cognitive improvement and concentration improvement (41). In terms of color preference, certain screening is carried out. Green, blue, red, purple, orange and yellow are the preferred order of people (34). Therapists who have taught autism courses for more than 2 years and have experience in children’s art education will teach the course, focusing on improving autistic children’s concentration, emotional control and simple affairs cognitive ability. In the healing activity, the therapist projected the traditional picture book onto the large screen, interacted with the participants or increased the visual attention of the students through a series of educational methods such as the therapist’s body language, music background, language expression, etc., and identified some prominent functions of a certain type of picture book in the treatment of children’s autism through the evaluation of the participants’ eye gaze, language communication, body communication, etc. (see Figure 7).

### 3.9. Intervention experiment procedure

In this study, a randomized controlled experiment was conducted to measure the intervention of the game group and the picture book group. Pre-test, intervention and post-test was adopted. In the pre-test and post-test, three methods were used to evaluate the autism symptoms of autistic children, including the expert evaluation of the Children’s Autism Rating Scale (CARS), the parent evaluation of the Children’s Autism Rating Scale (CARS) and the behavior skill evaluation of the autistic children’s Behavior Checklist (ABC). In order to control the additional variables, the experimental process ensures that the two groups have the same intervention time and the same interveners. At the same time, no additional game intervention is allowed for the experimenter. 2 groups (picture book group, game group) was used for all questionnaire data × 2 time (pre-test, post-test) repeated measurement ANOVA.

## 4. Research results

**Figure 8:**
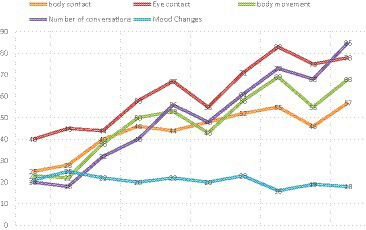
The behavioral record of somatosensory game intervention activities showed that in 10 intervention experiments, ASD children’s abilities improved, with significant improvements in communication and exercise, and stable emotional regions.

In 10 intervention experiments, the research group conducted data analysis on the behavior records of each intervention activity in the game group. The study found that ASD children showed significant improvement in all aspects. Although the overall amplitude of the game group changed, the improvement was significant, which may be related to the children’s behavior frequency, game planning plan, interaction plan, and other design. Among them, there was a significant increase in eye contact, dialogue frequency, and body movements, this indicates that the experimental group’s initiative in intervention experiments has improved and their focus has become increasingly high. The fourth item shows a decrease in the frequency of emotional changes, indicating that the child’s emotions are more stable. At the same time, the subjects participated in both the anterior and posterior tests, with a detachment rate of 0, indicating that the previous model design had a certain level of play ability (see Figure 8).

## 5. Discussion

### 5.1. Expert assessment of children’s autism rating scale (CARS)

The questionnaire measured 15 different aspects of the performance of children with autism, in which lower scores represented less severe symptoms of related autism, that is, better performance in related socialization; except for questions 3 (Emotional response), 9 (Proximate sensory response), and 11 (Verbal communication), in which higher scores represented better performance in related socialization.

**Figure 9:**
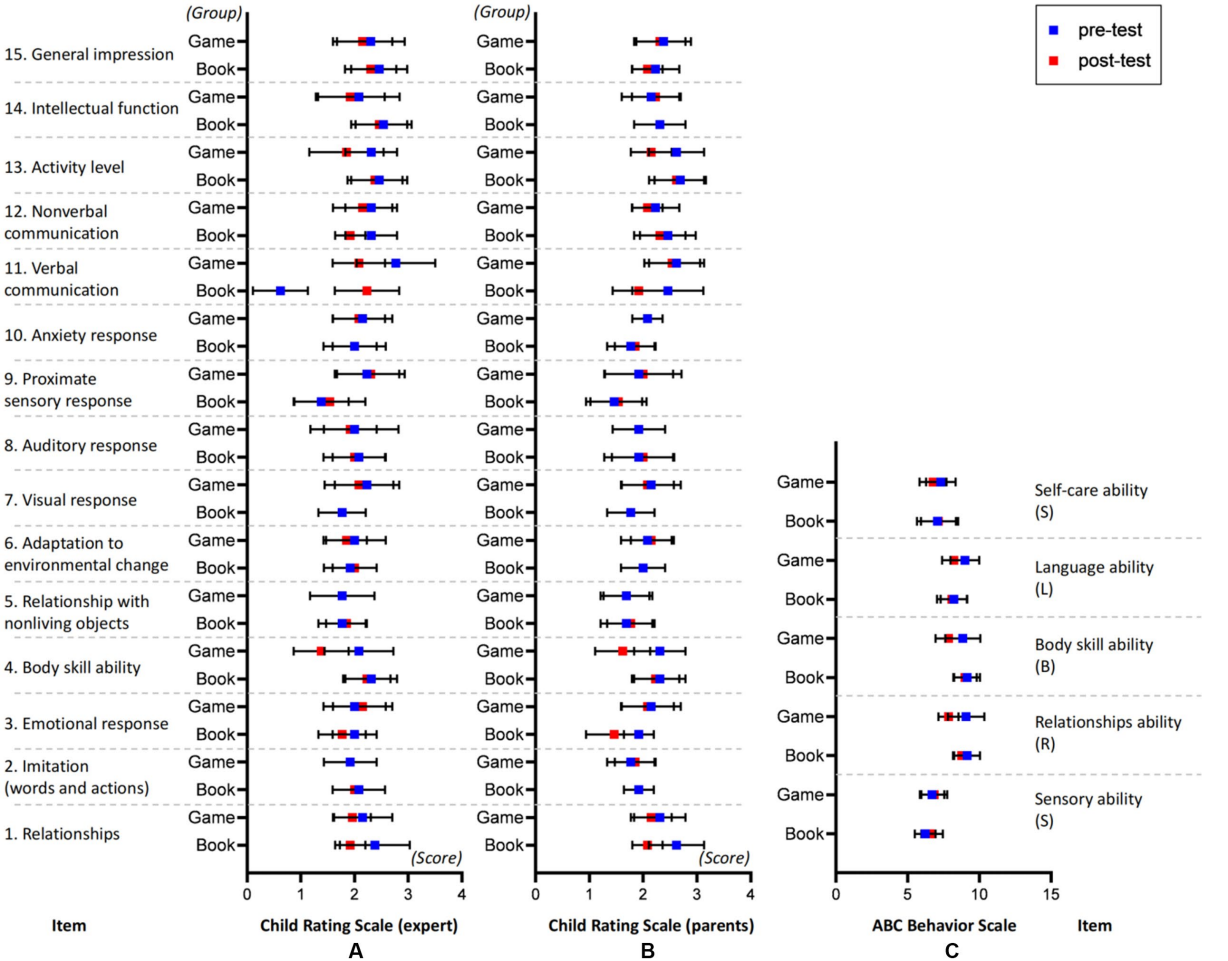
Somatosensory game group (referred to as GAME, experimental group) and traditional picture book group (referred to as BOOK, control group) scores on each scale in the pre-test and post-test. Comparisons of scores on **(A)** Child Rating Scale (Expert), **(B)** Child Rating Scale (Parents), and **(C)** ABC behavior scale.

The results of repeated measurement ANOVA are shown in Table 5 and Figure 9.

**Table 5:** Results of analysis of variance ANOVA of pre-test and post-test.

Variable	Picture book group		Somatosensory game group		*F*	*P*	*η^2^*
	Pre-test	post-test	Pre-test	Post-test			
N (male)	13 (12)	13 (12)	13 (12)	13 (12)			
Age (in years)	8.23	8.23	8.69	8.69			
Childhood Austism Rating Scale (CARS) (Expert)							
1. Relationships	2.38 (0.65)	1.92 (0.28)	2.15 (0.55)	1.96 (0.34)	3.000	0.096	0.111
2. Imitation (words and actions)	2.08 (0.49)	2.00 (0.00)	1.92 (0.49)	1.92 (0.49)	0.316	0.579	0.013
3. Emotional response	2.00 (0.41)	1.77 (0.44)	2.00 (0.58)	2.15 (0.55)	5.769	0.024	0.194
4. Body skill ability	2.31 (0.48)	2.23 (0.44)	2.08 (0.64)	1.38 (0.51)	16.000	0.001	0.400
5. Relationship with nonliving objects	1.77 (0.44)	1.85 (0.38)	1.77 (0.60)	1.77 (0.60)	1.000	0.327	0.040
6. Adaptation to environmental change	1.92 (0.49)	2.00 (0.41)	2.00 (0.58)	1.85 (0.38)	3.176	0.087	0.117
7. Visual response	1.77 (0.44)	1.77 (0.44)	2.23 (0.60)	2.08 (0.64)	2.182	0.153	0.083
8. Auditory response	2.08 (0.49)	2.00 (0.58)	2.00 (0.82)	1.92 (0.49)	0.000	1.000	0.000
9. Proximate sensory response	1.38 (0.51)	1.54 (0.66)	2.23 (0.60)	2.30 (0.63)	0.353	0.558	0.014
10. Anxiety response	2.00 (0.58)	2.00 (0.41)	2.15 (0.55)	2.08 (0.49)	0.316	0.579	0.013
11. Verbal communication	0.62 (0.51)	2.23 (0.60)	2.77 (0.73)	2.08 (0.49)	1.500	0.233	0.059
12. Nonverbal communication	2.31 (0.48)	1.92 (0.28)	2.31 (0.48)	2.15 (0.55)	1.742	0.199	0.068
13. Activity level	2.46 (0.52)	2.38 (0.51)	2.31 (0.48)	1.85 (0.69)	5.556	0.027	0.188
14. Intellectual function	2.54 (0.52)	2.46 (0.52)	2.08 (0.76)	1.92 (0.64)	0.353	0.558	0.014
15. General impression	2.46 (0.52)	2.30 (0.48)	2.30 (0.63)	2.15 (0.55)	0.000	1.000	0.000
Childhood Austism Rating Scale (CARS) (Parents)							
1. Relationships	2.62 (0.51)	2.08 (0.28)	2.31 (0.48)	2.15 (0.38)	4.688	0.041	0.163
2. Imitation (words and actions)	1.92 (0.28)	1.92 (0.28)	1.77 (0.44)	1.85 (0.38)	1.000	0.327	0.040
3. Emotional response	1.92 (0.28)	1.46 (0.52)	2.15 (0.55)	2.08 (0.49)	3.750	0.065	0.135
4. Body skill ability	2.31 (0.48)	2.23 (0.44)	2.31 (0.48)	1.62 (0.51)	16.000	0.001	0.400
5. Relationship with nonliving objects	1.69 (0.48)	1.77 (0.44)	1.69 (0.43)	1.69 (0.48)	0.316	0.579	0.013
6. Adaptation to environmental change	2.00 (0.41)	2.00 (0.41)	2.08 (0.49)	2.15 (0.38)	1.000	0.327	0.040
7. Visual response	1.77 (0.44)	1.77 (0.44)	2.15 (0.55)	2.08 (0.49)	1.000	0.327	0.040
8. Auditory response	1.92 (0.64)	2.00 (0.58)	1.92 (0.49)	1.92 (0.49)	1.000	0.327	0.040
9. Proximate sensory response	1.46 (0.52)	1.54 (0.52)	1.92 (0.64)	2.00 (0.71)	0.000	1.000	0.000
10. Anxiety response	1.77 (0.44)	1.85 (0.38)	2.08 (0.28)	2.08 (0.28)	1.000	0.327	0.040
11. Verbal communication	2.46 (0.66)	1.92 (0.49)	2.62 (0.51)	2.54 (0.52)	8.000	0.009	0.250
12. Nonverbal communication	2.46 (0.52)	2.31 (0.48)	2.23 (0.44)	2.08 (0.28)	0.000	1.000	0.000
13. Activity level	2.69 (0.48)	2.62 (0.51)	2.62 (0.51)	2.15 (0.38)	3.750	0.065	0.135
14. Intellectual function	2.31 (0.48)	2.31 (0.48)	2.15 (0.55)	2.23 (0.44)	1.000	0.327	0.040
15. General impression	2.23 (0.44)	2.08 (0.28)	2.38 (0.51)	2.31 (0.48)	0.353	0.558	0.014
ABC behavior scale							
Sensory ability (S)	6.23 (0.73)	6.70 (0.75)	6.70 (0.85)	6.85 (0.90)	1.655	0.211	0.065
Relationships ability (R)	9.15 (0.90)	8.77 (0.60)	9.08 (1.26)	7.85 (0.69)	5.223	0.031	0.179
Body skill ability (B)	9.15 (0.90)	9.00 (0.82)	8.85 (1.21)	7.85 (0.90)	9.553	0.005	0.285
Language ability (L)	8.23 (0.93)	8.08 (1.04)	9.00 (1.00)	8.23 (0.83)	3.282	0.083	0.120
Self-care ability (S)	7.08 (1.44)	7.15 (1.21)	7.31 (1.03)	6.77 (0.93)	5.818	0.024	0.195

Analytical adoption 2 groups × 2 time (pre-test, post-test) repeated measure analysis of variance was used in the analysis. The results showed that the interaction between groups and time was significant in indicators 3, 4, and 13 (*p*s < 0.05). Further simple analysis shows that: First of all, in the pre-test, there is no significant difference in the scores between the two groups, indicating the validity of random grouping. Secondly, in the comparison of pre and post test in index 3 (emotional response), after intervention, the score of the picture book group was significantly higher than that of the somatosensory game group. This indicator means the interaction with others at the emotional level, such as eye contact, expression response, gesture interaction, etc. The results showed that the intervention effect of picture book group was better than that of game group. In index 4 (body skill ability), after intervention, the score of the game group was significantly higher than that of the traditional picture book group. This indicator means the interaction with others at the level of physical expression, such as physical interaction, physical communication, etc. The results showed that the game group was more effective in the treatment of limb reactions. In indicator 13 (activity level), after intervention, the score of the game group was significantly higher than that of the traditional picture books group. This indicator means the trunk interaction that matches the content of the somatosensory interaction game, such as sports skills, balance ability, coordination ability and endurance. The results showed that somatosensory games could improve children’s activity level more than traditional picture books. In indicator 11 (language communication), after intervention, the picture book group scored significantly higher in language communication than the somatosensory game group. The meaning of this indicator is the response of children with autism in pronunciation, semantic expression, language application ability, and usage ability. The results indicate that traditional picture books have more ability to teach language communication than somatosensory games.

The comprehensive results show that traditional picture book therapy is superior to somatosensory game in identifying and enriching emotions and enhancing interpersonal response, while somatosensory game therapy has unique advantages in exercising physical ability and enhancing activity level.

Traditional picture book intervention uses traditional teaching methods, which are mainly taught by teachers and passively accepted by children. Therefore, children tend to recognize expressions by mechanical memory. In somatosensory games, children with autism tend to observe their own and others’ expressions spontaneously, and due to their slow learning ability, somatosensory games have poor efficacy in emotion recognition and response for children. Somatosensory game intervention mainly consists of interactive game activities. Besides traditional teaching, there are also physical interaction methods such as jumping, waving, and selecting activities. Therefore, it has a good effect in terms of training body coordination ability and enhancing activity level.

### 5.2. Parent assessment of children’s autism rating scale (CARS)

Analytical adoption 2 groups × 2 time (pre-test, post-test) repeated measure analysis of variance was used in the analysis. The results showed that the interaction between groups and time was significant in indicators 1, 4, and 11 (*p*s < 0.05). Further simple analysis shows that: First of all, in the pre-test, there is no significant difference in the scores between the two groups, indicating the validity of random grouping. Secondly, in the comparison of pre and post test, in index 1 (interpersonal relationship), after intervention, the score of picture book group was significantly higher than that of somatosensory game group. The meaning of this indicator is the breakthrough performance of autistic children in emotional disorders, such as obtaining profitable social reactions, being grateful for others’ help, improving their ability to participate in collaborative team games, showing understanding and reflecting on others’ emotions, etc. The results show that traditional picture books have more positive guidance ability to identify interpersonal relationships. In index 4 (body skill ability), after intervention, the score of the game group was significantly higher than that of the traditional picture book group. This index implies that somatosensory games have higher efficacy in the healing of physical operation ability. In index 11 (language communication), after intervention, the picture book group scored significantly higher than the somatosensory game group. The meaning of this index is the reaction of autistic children in voice, semantic expression, language application ability and use ability. The results show that traditional picture books are more capable of teaching language communication than somatosensory games.

The comprehensive results show that traditional picture book therapy is superior to somatosensory games in interpersonal relationship guidance and language communication learning, while somatosensory game therapy has unique advantages in physical coordination.

Traditional picture book teaching can directly guide autistic children in language and teach them how to deal with interpersonal relationships, while somatosensory games are personal experiences in activities and spontaneous language communication. Due to the limited intervention process, spontaneous learning effect is not significant.

### 5.3. Comparison of expert and parent assessment of children’s autism rating scale (CARS)

It was also noted that compared with the results of the expert, the results of the parent showed that the interaction between groups and time was marginal significant in indicators 3 (emotional response) and 13 (activity level) (*p*s = 0.065). Further simple analysis showed that the trend of the results was consistent with the children rating scale (experts), that is, after intervention, the emotional response score of the picture book group was significantly higher than that of the somatosensory game group, and the body skill ability score of the game group was significantly higher than that of the traditional picture book group.

By comparing the two results of the two group, it was found that the overall trend was consistent, that is, the advantage of traditional picture book therapy lies in identifying and enriching emotions and learning language communication, while the advantage of somatosensory games lies in exercising body coordination and enhancing activity level.

### 5.4. Autistic children’s behavior checklist (ABC)

Analytical adoption 2 groups × 2 time (pre-test, post-test) repeated measure analysis of variance was used in the analysis. The results showed that the interaction between groups and time was significant in index 2, 3, and 5 (*p*s < 0.05). Further simple analysis shows that: First of all, in the pre-test, there is no significant difference in the scores between the two groups, indicating the validity of random grouping. Secondly, in the comparison pre and post test, in index 2 (communicative ability), the score of the game group after intervention was significantly higher than that of the traditional picture book group. This indicator means the performance of social interaction with others, such as social gaze, attachment, partnership, participation in games, etc. The number of eye contact after somatosensory game intervention is significantly higher than that of traditional picture book intervention, and the sense of participation in games becomes stronger, and some students begin to accept team games. In index 3 (motor ability), after intervention, the motion ability score of the game group was significantly higher than that of the traditional picture book group. This index means that the level of physical activity was enhanced after intervention of the somatosensory game. In index 5 (self-care ability), after intervention, the score of somatosensory games is better than that of traditional picture books. The meaning of this index refers to the practice life ability of autistic children. Through the step-by-step action demonstration of somatosensory interactive games, the process of “doing” in children’s self-care skills in life has been significantly improved.

The comprehensive results show that somatosensory game therapy has unique advantages in the improvement of the behavior of children with autism, which is reflected in the increase of the number of eye contact, the enhancement of the level of physical activity, and the enhancement of dialogue and communication and the control of emotional fluctuations.

This project is from the perspective of art therapy, exploring the key factors that can enhance ASD children’s abilities in interpersonal communication, emotional management, social disorders, and motor skills through art works. Due to the more intuitive nature of the works, the key factors presented in the works can serve as a reference for designers when designing similar products. Psychological research serves as an important support for the team, and its data results will validate the healing function of the design work: the picture book group outperforms the game group in terms of communication ability, physical movement, and self-care ability. These advantages may stem from certain aspects of somatosensory interaction game design: (1) Subitem interventions can be conducted on ASD children, including social ability, motor ability, cognitive education, and intellectual enhancement. (2) Use relatively simple guidance and reasonable audio-visual feedback in interaction to ensure that ASD children have enough interest and patience. (3) Simple Icon design is more effective for ASD children’s game understanding. (4) Design a color system and matching method that suits the preferences of ASD children. Therefore, these design factors are considered as the core factors of play therapy for children with ASD.

## 6. Conclusion

Based on the integration of art and psychology, this study designed two somatosensory interactive games suitable for ASD children. Through a randomized scientific control, it was found that the game group was more effective than the picture book group in terms of physical interaction, communication, facial expression response, and eye contact. The research also found that the step-by-step action demonstration of somatosensory interactive games significantly improved the self-care skills of ASD children and responded better to environmental changes, fully validated the effect of somatosensory interaction games on children with ASD. The final research concluded that the core factors affecting play therapy in practice were subentry intervention, interaction and feedback, icon design and color matching system through artistic analysis. In future work, it is expected to continue to conduct in-depth research on art therapy tools to broaden the media for intervention in children with autism and develop family based interventions. In terms of knowledge that needs to be deepened, the various abilities and related experiences of ASD children when using products can be extended to other treatment disorders, such as attention deficit disorder or hyperactivity disorder.

## 7. Consequence

This study received support from local special education schools, which became a collaborative research base for the team and provided long-term funding. Healing activities have officially been included in the regular curriculum of the school.

## 8. Practical significance

Our research has designed a reliable and effective somatosensory game therapy, and the experimental results have verified the unique positive significance of somatosensory game therapy in the intervention treatment of autistic children, that is, compared with traditional picture book therapy, somatosensory game therapy has stronger intervention advantages in physical activity, eye contact and interaction, emotional control, etc. More importantly, based on the design of somatosensory game therapy, the game can also be developed into corresponding software, which can prolong the healing time of autistic children. Because the games have the healing function, autistic children can frequently use the games to improve their social communication ability, language expression ability, physical movement ability, etc. Therefore, somatosensory game therapy can become an important complementary means of traditional autism intervention therapy.

## 9. Limitations and prospects

This study still has some limitations. First of all, this study belongs to a small sample study, and the representativeness of the sample may be limited, so we need to be cautious in generalizing the conclusions. Moreover, if we want to develop software with wide applicability based on this game design, we need to consider more empirical research on the basis of large samples in the future. Secondly, this study only studied the effect of somatosensory game therapy on children with mild or moderate autism, but did not evaluate its intervention effect on children with severe autism. In the future, targeted intervention programs for children with severe autism should be considered. Finally, this study verified that somatosensory game therapy and traditional picture books therapy have their own advantages. In the future, a comprehensive intervention plan containing multiple interventions should be considered to improve the intervention effect on autistic children.

## Data availability statement

The datasets presented in this article are not readily available because our data set involves many personal privacy, moral and ethical issues. And parents do not agree to disclose the privacy of their children. Requests to access the datasets should be directed to 316892335@qq.com.

## Ethics statement

The studies involving humans were approved by the Ethics Committee of the Maanshan Psychological Society. The studies were conducted in accordance with the local legislation and institutional requirements. Written informed consent for participation in this study was provided by the participants’ legal guardians/next of kin. Written informed consent was obtained from the individual(s) for the publication of any potentially identifiable images or data included in this article.

## Author contributions

SH and ZW: experimental design and thesis writing. CX: thesis writing and interactive game design. GG, JY, and LY: experimental test and analysis. All authors provided critical feedback, reviewed, and approved the final manuscript.
